# Prevalence and management of patients using medication targeting obstructive lung disease: A cross-sectional study in primary healthcare in Greenland

**DOI:** 10.3402/ijch.v72i0.20108

**Published:** 2013-02-28

**Authors:** Sequssuna Olsen, Dorte Ejg Jarbøl, Mette Kofoed, Kim Abildskov, Michael Lynge Pedersen

**Affiliations:** 1 Queen Ingrids Health Care Centre, Nuuk, Greenland; 2 Research Unit of General Practice, Institute of Public Health, University of Southern Denmark, Odense, Denmark; 3 Greenland Centre for Health Research, Nuuk, Greenland

**Keywords:** Greenland, Inuit, Arctic, chronic obstructive lung disease, primary healthcare, quality

## Abstract

**Objective:**

The aim of this study was to estimate the prevalent use of drugs targeting obstructive lung diseases among adults aged 50 or above in Greenland and to assess the use of spirometry testing among these medication users.

**Study design:**

Observational cross-sectional study based on reviews of electronic medical records.

**Methods:**

The study was performed in the 6 largest primary healthcare clinics in Greenland, representing approximately 67.0% of the population in Greenland. Adults aged 50 years or above, who had at least one electronically prescribed drug targeting obstructive lung diseases within a 15-month time interval, were identified. We assessed whether a spirometry test was registered in their medical records within previous 2- and 4-year periods.

**Results:**

A total of 565 persons were identified. This corresponds to a prevalent medication use of 6.1% (565/9,023) among adults aged 50 years or above. Among these medication users, 14.1% (80/565) had a spirometry test performed within 2 years. Within the 4-year period this increased to 17.9% (101/565).

**Conclusion:**

The use of medication targeting obstructive lung diseases in Greenland among adults aged 50 years or above is common. However, spirometry testing among medication users is low and interventions aiming to increase focus on spirometry testing should be integrated in the primary healthcare system.

Obstructive lung diseases like asthma and chronic obstructive pulmonary disease (COPD) are common chronic conditions, and international guidelines recommend spirometry as the gold standard for diagnosing chronic obstructive lung disease (1, 2).

Adults being treated with medication targeting obstructive lung disease should initially have spirometry performed to distinguish between their symptoms being due to obstructive lung disease or other illnesses, also giving respiratory symptoms. Guidelines recommend that patients with obstructive lung disease are monitored with spirometry testing, enabling adequate medication adjustments. Both the Global Initiative for Asthma (GINA) and the Global Initiative for Chronic Obstructive Lung Disease (GOLD) guidelines recommend spirometry at least annually, although asthma patients, who are monitored according to symptom severity, often need more frequent testing (1).

Several studies have demonstrated a gap between recommendations and actual practice in the primary healthcare system. Patients are diagnosed with asthma and COPD without spirometry testing (3–5) and they are not monitored adequately (6). Studies have also shown that patients are prescribed medication targeted at obstructive lung disease without spirometry testing (7, 8) and without any diagnosis registered (9).

Greenland has a high prevalence of smokers (10) and we hypothesise that the prevalence of obstructive lung disease, especially COPD, is high due to this risk factor. The prevalence of obstructive lung disease in Greenland is, however, unknown and regional differences are not assessed. Also, there are no studies assessing what proportion of the population is using medication targeting obstructive lung disease or to what extent these medication users are monitored with spirometry.

The aim of this study was to estimate the prevalent use of medication targeting obstructive lung disease among adults aged 50 years or above in different towns in Greenland and to assess the use of spirometry testing among these medication users.

## Material and method

### Setting

Greenland is a big country covering an area of over 2 million km^2^ and is thus the world's largest island. The country is sparsely populated with approximately 56,000 inhabitants, mostly of Inuit origin, spread over a wide area along the coast. There are 18 towns and around 60 minor settlements (11, 12).

All electronically generated prescriptions are produced at the local healthcare centre (13). Patients with obstructive lung disease are seen and controlled in their local healthcare centre. Patients with more severe obstructive lung disease can be referred to travelling specialists in internal medicine. However, the medical records from these consultations are included and kept in the Electronic Medical Record (EMR).

### Study population

The study was designed as a cross-sectional study of the population in the 6 largest towns in Greenland, representing 67.0% of the entire population. Observational data were collected retrospectively through the prescription module part of the EMR system, implemented in the Greenland primary healthcare system. The criteria for inclusion were adults aged 50 or above having medication targeting obstructive lung disease prescribed electronically within a period of 15 months prior to the time of data extraction. Drugs for obstructive lung disease were defined as drugs with the Anatomical Therapeutic Chemical (ATC) code R03. The electronic identification was done for each of the 6 healthcare clinics from November 2010 to April 2011. Information about age and gender was obtained for the study population.

### Spirometry testing

Among all users of medication targeting obstructive lung disease, a further review of their medical records was performed. We registered if a spirometry measurement was recorded in the EMR within the previous 2 (after 1 January 2009) or 4 years (after 1 January 2007. We also registered if the diagnosis of COPD was recorded since spirometry is mandatory in the diagnosis of COPD. No information about diagnosis of other obstructive lung diseases were recorded in this study.

### Analysis and statistics

The prevalent use of medication targeting obstructive lung disease among adults aged 50 or above was estimated, using the background population as of 1 January 2011 (14). The proportion of medication users having a spirometry test performed within a 2- and 4-year period was recorded.

Chi-square tests were used to compare frequencies between the 6 towns with regard to medication use and spirometry registration. P-value at 0.05 was used as a level of significance. Estimates were calculated with 95% confidence intervals.

## Results

The study included the 6 largest towns in Greenland, representing 67% (37,940/56,615) of the entire population of Greenland. A total of 9,023 persons aged 50 or above were living in the towns (4,993 males and 4,030 females).

A total of 565 people of the population aged 50 or above received an electronic prescription for R03 medication in the 15 month time interval. This corresponds to a prevalent medication use of 6.3% (95% CI: 5.8–6.8). The prevalence differed significantly between the 6 towns (P<0.01; Table I).

**Table I:** Prevalence of use of medication targeting obstructive lung disease among subjects aged 50 years or above in 6 towns in Greenland and spirometry testing among these medication users

Town	Nuuk	Sisimiut	Ilulissat	Qaqortoq	Maniitsoq	Aasiaat	Total	P*
Prevalence of medication use within a 15-month interval	6.9% (258/3,725)	6.0% (87/1,452)	5.8% (68/1,171)	5.5% (51/935)	4.2% (39/920)	4.6% (62/820)	6.3% (565/9,023)	<0.01
Spirometry registered within 2 years	13.6% (35/258)	4.6% (4/87)	30.8% (21/68)	11.8% (6/51)	2.6% (1/39)	21% (13/62)	14.1% (80/565)	0.01
Spirometry registered within 4 years	14% (36/258)	9.2% (8/87)	33.8% (23/68)	15.7% (8/51)	2.6% (1/39)	40.3% (25/62)	16.3% (101/565)	<0.01

* Chi-square tests.

The use of medication targeting obstructive lung disease varied significantly between gender (P<0.001); among females 9% (95% CI: 8.2–9.9; 365/4,030) received R03 medication, whereas among males only 4% (95% CI: 3.5–4.5; 200/4,993) received this type of medication.

Among medication users, the percentage of patients having a spirometry test performed within 2 and 4 years was 14% (80/565) and 18% (101/565), respectively. The proportion of medication users with a spirometry test performed varied significantly between the 6 towns (P<0.01; Table I).

In total, 214 (145 females and 69 males) had the COPD diagnosis registered in their medical record, corresponding to 38% (214/565) of persons with a prescription for R03 medication. Among the patients with the COPD diagnosis, 25% (54/214) and 32% (68/214) had a spirometry test performed within 2 and 4 years, respectively. The proportion of patients with asthma was not accessed in this study.

## Discussion

The prevalence of patients treated with medication for obstructive lung disease among adults aged 50 years or above in Greenland was 6%, indicating a frequent use. However, only 18% of these medication users had spirometry performed within 4 years, thus demonstrating a limited use of spirometry in the primary healthcare system in Greenland.

### Prevalence in other studies

A study conducted by Montes et al. (7) examined the use of bronchodilators and corticosteroids in 5 Latin American cities. Spirometry was conducted, and it was registered if subjects had a history of spirometry testing. A prevalence at 6.5% of the patients reported use of any bronchodilator or corticosteroid in the previous 12 months. Among medication users, 37.5% (135/360) reported a history of spirometry testing. Our study demonstrated an almost identical prevalence of patients treated with medication targeting obstructive lung disease but a lower spirometry rate among medication users.

The data from Montes et al. suggest a similar widespread practice of drug prescribing for obstructive lung disease based on symptoms and not properly evaluated with spirometry testing. Montes et al. also demonstrated that the majority of patients receiving bronchodilators and/or corticosteroids had no airway obstruction when tested with spirometry. Whether similar findings exist in Greenland would be relevant to investigate.

Our study showed that the prevalence of patients prescribed drugs for obstructive lung disease differed with regard to gender, with a higher prevalence among women. Similar results were seen in the study by Montes et al. where the difference may be partly explained by a well-known higher prevalence of asthma among adult women than among adult men (15) and partly by more frequent contact to the primary healthcare system for women compared to men in Greenland (16).

The prevalence of patients prescribed drugs for obstructive lung disease within the 6 towns in Greenland varied considerably. The reason for this is unclear. It could be caused by a difference in the symptomatology, the frequency of use of primary healthcare, the occurrence of obstructive lung diseases or a difference in the awareness of the disease in the 6 different healthcare centres in Greenland. Our study showed that among patients, who had been prescribed medication for obstructive lung disease, 38% had the diagnosis of COPD registered in their medical record. Among patients with COPD, only 32% had spirometry testing within 4 years. Similar findings were seen in the PLATINO study, where 34% of the patients with COPD had been tested with spirometry prior to the diagnosis (17). In a Swedish study conducted by Arne et al. (5), only 59% of the patients with COPD diagnosis had spirometry data in their medical records. It suggests a widespread underutilisation of spirometry among patients with COPD.

In a Danish study conducted by Lange et al. (6), the quality of the COPD care in general practice in Denmark was assessed. In total 124 general practitioners participated in the 2 cross-sectional surveys. The study examined the quality of COPD management in general practice before and after the doctors had participated in a specific COPD education programme. The results showed an improvement in registered spirometry tests from 53 to 71%. Our study showed that a spirometry test was registered in 25% of patients with a COPD diagnosis, demonstrating an area for improvement in the primary healthcare system in Greenland.

### Strengths and weaknesses

The strength of this study is that it covered 67% of the entire population of Greenland. The data recorded from primary healthcare clinics in the 6 towns were used to identify the prevalence of users of drugs targeting obstructive lung disease and the use of spirometry tests. The benefits of the EMR system included the possibility of using a standardised search tool and the possibility of including all patients with contact to the healthcare system in the 6 towns. With this method, it is possible to monitor and correlate the outcomes for further reinvestigation.

A possible disadvantage of the method is that some clinicians issue handwritten prescriptions, not using the EMR, and we may therefore underestimate the prevalence of medication users. However, it is assumed that handwritten prescriptions are infrequently used, because the EMR system is incorporated in the physicians’ daily contact with the patient.

It cannot be excluded that some patients have the drugs prescribed without filling in the prescription. This would potentially overestimate the prevalence of medication users. However, primary non-compliance is considered small (18), and furthermore, prescription medication in the Greenlandic healthcare system is free of charge and delivered directly at the healthcare clinic.

A clear limitation in this study is that the International Classification of Primary Care (ICPC-2) is not used on a regular basis in Greenland. Thus, we had to rely on diagnosis recorded in the text. For this reason, we only looked at the diagnosis of COPD where spirometry testing is mandatory and consequently we do not have information about other obstructive lung diseases.

With regard to underestimating spirometry testing it is possible that some clinicians do not register spirometry testing. However, physicians are required by law to keep medical records. Another cause of possible underestimation of spirometry testing could have arisen because we retrospectively assessed if spirometry was performed when medication was prescribed. For first-time users of obstructive lung disease medication, a prospective assessment could have captured more spirometry testing. It is, however, assumed only to be the case for a minor group of patients, and therefore not considered to be a significant problem.

## Conclusion and perspective

The use of drugs for obstructive airway diseases in Greenland among adults aged 50 years or above is common. However, the use of spirometry testing among medication users is low, and interventions aiming to increase focus on spirometry testing should be integrated into the primary healthcare system.

The population of Greenland has a high prevalence of cigarette smokers (10) and therefore it must be assumed that the healthcare system faces a major challenge in managing the burden of COPD. Substantial improvements must be considered in order to diagnose, treat and monitor the disease.

Similar to the suggestions of the study on treatment and monitoring of hypertension in Greenland (19), a national strategy is recommended based on guidelines, use of electronic drug prescriptions, spirometry testing and smoking status recording annually in order to improve the quality of care.
